# Prediction of EGFR mutations in non-small cell lung cancer: a nomogram based on ^18^F-FDG PET and thin-section CT radiomics with machine learning

**DOI:** 10.3389/fonc.2025.1510386

**Published:** 2025-04-02

**Authors:** Jianbo Li, Qin Shi, Yi Yang, Jikui Xie, Qiang Xie, Ming Ni, Xuemei Wang

**Affiliations:** ^1^ Department of Nuclear Medicine, The Affiliated Hospital of Inner Mongolia Medical University, Hohhot, China; ^2^ Department of Nuclear Medicine, Division of Life Sciences and Medicine, The First Affiliated Hospital of USTC, University of Science and Technology of China, Hefei, China

**Keywords:** nomogram, non-small cell lung cancer, PET/CT, machine learning, epidermal growth factor receptor

## Abstract

**Background:**

This study aimed to develop and validate radiomics-based nomograms for the identification of EGFR mutations in non-small cell lung cancer (NSCLC).

**Methods:**

A retrospective analysis was performed on 313 NSCLC patients, who were randomly divided into training (n = 250) and validation (n = 63) groups. Radiomic features were extracted from ^18^F-fluorodeoxyglucose positron emission tomography (^18^F-FDG PET) and thin-section computed tomography (CT) scans. After selecting optimal radiomic features, four machine learning algorithms, including logistic regression (LR), random forest (RF), support vector machine (SVM), and extreme gradient boosting (XGBoost), were used to develop and validate radiomics models. A combined model, incorporating the Rad score from the best performing radiomics model with clinical and radiological features, was then formulated. Finally, the integrated nomogram was generated. Its predictive performance and clinical utility were evaluated using receiver operating characteristic curves, calibration curves, and decision curve analysis.

**Results:**

Among the radiomics models, the RF model showed the best performance with AUCs of 0.785 (95% CI, 0.726-0.844) and 0.776 (95% CI, 0.662-0.889) in the training and validation groups, respectively. The AUCs of the clinical and radiological models in both groups were 0.711 (95% CI, 0.645-0.776) and 0.758 (95% CI, 0.627-0.890), and 0.632 (95% CI, 0.564-0.699) and 0.677 (95% CI, 0.531-0.822), respectively. The combined model achieved the highest AUCs of 0.872 (95% CI, 0.829-0.915) and 0.831 (95% CI, 0.723-0.940) in the training and validation groups, respectively. The DeLong test confirmed the superiority of the combined model over the other three models. Both the calibration curve and the DCA indicated that the radiomics nomogram was consistent and clinically useful.

**Conclusions:**

Radiomics combined with machine learning and based on ^18^F-FDG PET/CT images can effectively determine EGFR mutation status in NSCLC patients. Radiomics-based nomograms provide a non-invasive and visually intuitive prediction tool for screening NSCLC patients with EGFR mutations in a clinical setting.

## 1 Introduction

In 2024, lung cancer will remain the leading cause of cancer-related deaths worldwide (1). Non-small cell lung cancer (NSCLC) accounts for approximately 80-85% of all lung cancers (2). Unfortunately, most NSCLC patients are diagnosed at an advanced stage, resulting in a poor prognosis (3). With the advent of precision medicine and personalized treatment strategies, the paradigm of targeting the epidermal growth factor receptor (EGFR) with tyrosine kinase inhibitors (TKIs) has become the standard of care for advanced NSCLC. This approach has significantly improved progression-free survival and overall survival in patients with EGFR mutations (4, 5). However, resistance to TKIs inevitably develops over time (6). The National Comprehensive Cancer Network (NCCN) guidelines recommend molecular detection of EGFR mutations in patients with advanced or metastatic NSCLC (7). Therefore, rapid and accurate identification of EGFR mutations is of paramount importance for tailoring individualized treatment plans.

Currently, gene mutation detection relies primarily on histological samples from primary or metastatic lesions. Invasive procedures often yield limited tissue or cell samples that may not accurately represent the overall tumor profile or capture intra- and inter-tumor heterogeneity. In addition, approximately 5% to 20% of patients with advanced NSCLC cannot undergo molecular genetic testing using histological samples (8). Liquid biopsy has emerged as a novel method to assess EGFR mutation status. Although it offers convenience, speed and affordability, its sensitivity and stability remain suboptimal (9). Therefore, there is an urgent need to develop non-invasive, simple, rapid and reliable techniques for detecting gene mutations.

Phenotypic analysis by imaging is a promising non-invasive method for predicting EGFR mutations. Previous studies have shown that CT signs, including ground glass composition, air-bronchial sign, vacuole sign and pleural indentation sign, correlate with EGFR mutations (10–12). However, these CT signs rely on subjective visual assessment and lack quantification. ^18^F-FDG PET/CT, recognized as a molecular imaging technique for tumors, has become an integral part of the clinical management of NSCLC (7). While EGFR mutation may influence FDG uptake via the NADPH oxidase 4 (NOX4)/reactive oxygen species (ROS)/glucose transporter protein 1 (GLUT1) axis (13), the predictive value of ^18^F-FDG PET/CT metabolic parameters - such as the maximum standard uptake value (SUVmax), mean standard uptake value (SUVmean), total lesion glycolysis (TLG) and metabolic tumor volume (MTV) - remains controversial in the context of EGFR mutation status (14).

Radiomics offers a departure from traditional image analysis by transforming medical images into high-dimensional, mineable data through the high-throughput extraction of quantitative features. This approach has shown significant potential in tumor diagnosis, treatment evaluation and prognosis prediction (15). While numerous machine learning models based on radiomics features have been reported to identify EGFR mutation status in NSCLC patients (16–18), most radiomics studies have typically used a single modelling method, which may affect predictive outcomes. To improve the accuracy of radiomics in predicting EGFR mutations, our study used different machine learning algorithms to build multiple models. Furthermore, we constructed a combined model that integrates PET/CT radiomics with clinical and radiological features to optimize prediction efficiency. A radiomics-based nomogram was then developed to predict EGFR mutation status.

## 2 Materials and methods

### 2.1 Patients

This study was conducted in accordance with the Declaration of Helsinki, as revised in 2013, and was approved by the Ethics Committee of The First Affiliated Hospital of University of Science and Technology of China (approval number 2023-RE-018). The data are anonymous, and the requirement for informed consent was therefore waived. 313 patients were retrospectively analyzed from January 2015 to June 2021. Inclusion criteria included: NSCLC diagnosis confirmed by surgical or puncture biopsy with subsequent EGFR gene detection; ^18^F-FDG PET/CT scan within one month prior to treatment; and no history of other malignancies. Exclusion criteria included: prior antitumor treatment prior to the PET/CT scan; poor image quality due to significant respiratory or motion artefacts, or unclear tumor boundaries making it difficult to delineate the lesion volume of interest (VOI); a lesion VOI of less than 1.0 cm^3^, which could introduce a partial volume effect; lesions identified as ground glass nodules (GGNs) or with an SUVmax < 2.5; multiple lung cancer lesions (≥ 2); and incomplete clinical or imaging data. Of the 313 patients analyzed, 123 were identified as having a wild-type EGFR genotype, while 190 had EGFR mutations. These patients were randomly assigned in an 8:2 ratio to a training group (n = 250) and a validation group (n = 63). Both clinical and radiological features were meticulously documented.

### 2.2 EGFR mutation detection

Tumor tissue samples were obtained either by either surgical resection or biopsy. EGFR mutation status was analyzed using the human EGFR gene mutation detection kit provided by Wuhan Friends Medical Technology Co, Ltd, China. Mutations in EGFR exons 18, 19, 20 and 21 were identified using the real-time PCR/amplification retardation mutation system (RT-PCR/ARMS). PCR analysis was performed on the PRISM 7500 system from Applied Biosystems, Inc. Experienced pathologists with over a decade of experience interpreted and confirmed both histological findings and EGFR mutation results.

### 2.3 Image acquisition

Prior to the scan, patients were required to fast for more than 6 hours and maintain a blood glucose level of less than 11.10 mmol/L. They were then administered ^18^F-FDG at a dose of 3.7-7.4 MBq/kg. After a rest period of approximately 60 ± 10 minutes, patients underwent a PET/CT scan using a Biograph 16HR PET/CT scanner (SIEMENS, Germany). A low-dose CT scan was performed first, followed by a PET scan. The PET acquisition used a three-dimensional mode over 6-8 beds, with each bed taking approximately 2 minutes. PET images, attenuated with CT data, were reconstructed using the ordered subset expectation maximization method (3 iterations, 24 subsets, and a 4 mm full width at half maximum). To obtain more detailed morphological information, a breath-hold thin-section CT scan was performed immediately after the PET/CT scan. The acquisition parameters for this scan were set to a voltage of 120 kV, a current of 200 mA, a pitch of 1.15, a collimator width of 0.75 mm, a reconstruction slice thickness of 0.625 mm, and a matrix of 512 × 512.

### 2.4 Tumor segmentation

In accordance with the Image Biomarker Standardization Initiative (IBSI) (19), the volume of interest (VOI) was delineated by two nuclear medicine physicians with more than 10 years of experience using the TrueD toolkit on the Syngo via workstation (version VB10B, SIEMENS). The VOI included areas of necrosis, hemorrhage and calcification while excluding normal lung tissue, atelectasis or surrounding tumor inflammation. For PET images, physicians initially set the SUVmax threshold at 40% and then used the adaptive brush tool for semi-automated 3D segmentation to ensure that the VOI visually encompassed the entire primary tumor (20). Metabolic volume parameters such as SUVmax, SUVmean, peak standard uptake value (SUVpeak) and MTV were calculated automatically on the same post-processing workstation. TLG was derived using the formula TLG = SUVmean × MTV. For thin-section CT images, 3D semi-automated segmentation software was used, based on the region growth segmentation method (taking into account homogeneity and grey level differences), followed by manual layer-by-layer adjustments. Maximum tumor diameter (MTD) and gross tumor volume (GTV) were determined automatically. Thin-section CT radiological features included lesion size (MTD and GTV), lobulation, spiculation, pleural indentation, vacuole sign, cavity sign, vascular convergence, air bronchogram and calcification. To evaluate the reliability and reproducibility of radiomics features by calculating intra- and interobserver intraclass-correlation coefficient (ICC). Both physicians were blinded to patient pathology results and EGFR mutation status. Physician 1 and Physician 2 randomly selected 72 patients from the enrolled group to draw VOI from PET and thin-section CT images, with Physician 1 drawing again after 2 weeks. VOI segmentation of the remaining cases was performed by Physician 1.

### 2.5 Feature extraction and selection

Radiomics features were extracted using the PyRadiomics package in Python (version 3.0.1) (21). To minimize errors in the image data acquisition process, all images were normalized and resampled to a uniform resolution of 1 × 1 × 1 mm^3^ by interpolation prior to feature extraction. From the original images, the radiomics features included 14 shape-based, 18 first-order statistics, 24 grey level co-occurrence matrix (GLCM), 16 grey level run length matrix (GLRLM), 16 grey level size zone matrix (GLSZM), 14 grey level dependence matrix (GLDM), and 5 neighborhood grey tone difference matrix (NGTDM) features. The morphological features were only extracted from the original image. In addition to the original image features, we also extracted features after wavelet and local binary pattern (LBP) filtering to capture more efficient attributes. Wavelet filtering resulted in eight decomposed images representing all combinations of high-pass (H) or low-pass (L) filters applied in three dimensions, namely: wavelet-HHH, wavelet-HHL, wavelet-HLH, wavelet-HLL, wavelet-LHH, wavelet-LHL, wavelet-LLH and wavelet-LLL. A total of 944 PET and 944 CT radiomics features were extracted. Initial refinement using Pearson’s correlation test eliminated 1,447 features with a correlation coefficient |r| ≥ 0.90, leaving 441 features. Subsequent analysis of variance (ANOVA) identified the top 60 features with the most significant variance. To ensure feature reliability and reproducibility during segmentation, only 43 features with an ICC greater than 0.9 were retained. Finally, to mitigate overfitting, the least absolute shrinkage and selection operator (Lasso) algorithm combined with 10-fold cross-validation was used for optimal selection of the radiomics feature subset. Baseline clinical variables including age, sex, smoking status, tumor location (central/peripheral), lung lobe, clinical staging, TNM staging, tumor indicators (CEA, SCC and CYFRA21-1), histological subtypes and 13 other variables were collected, yielding a total of 33 clinical features. Radiological features included thin-slice CT features and PET features. Radiological features included the above thin-slice CT features and PET features, resulting in a total of 23 variables. Pearson correlation analysis, Mann-Whitney U test, ANOVA analysis, and 10-fold cross-validated Lasso regression model were used to screen clinical variables and radiological features.

### 2.6 Construction and validation of the model

**Figure 1:**
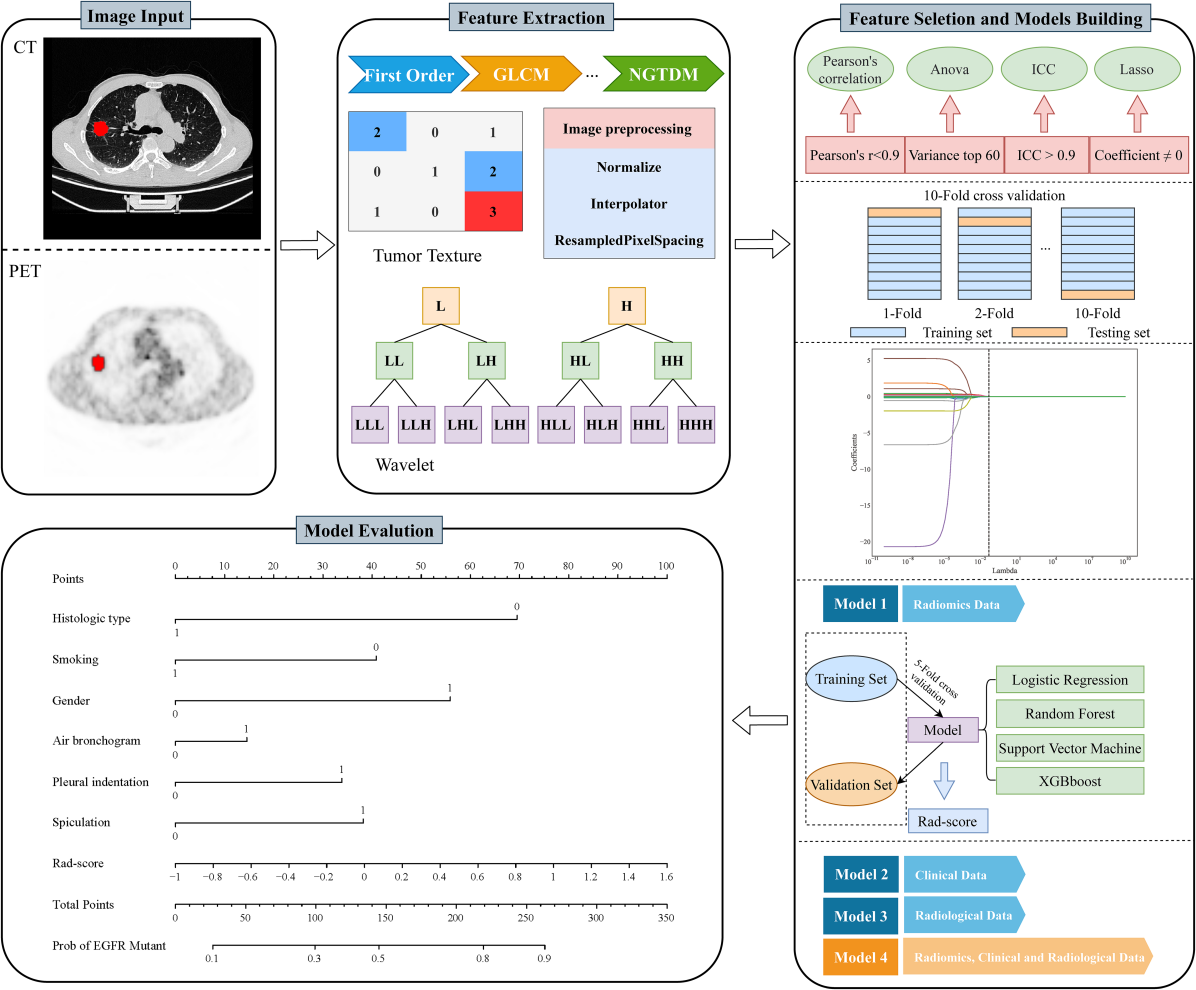
The workflows of this study.

Machine learning model constructing and performance evaluation were performed using Python. Four machine learning algorithms - logistic regression (LR), random forest (RF), support vector machine (SVM) and extreme gradient boosting (XGBoost) - were used to construct the radiomics models. A 5-fold cross-validation was implemented to ensure model robustness. The predictive ability of each algorithm was primarily assessed using the area under the curve (AUC) from receiver operating characteristic (ROC) curve analysis. The model with the highest AUC was considered the optimal radiomics model, from which the radiomics score (Rad score) was derived. Similarly, clinical variables and radiological features were screened and models were constructed using logistic regression. To combine radiomics, radiological and clinical features, we developed a combined model and assessed its performance. The identified clinical and radiological features, together with the Rad score, were then used to construct the nomograms. The goodness of fit of the nomograms was assessed using the calibration curve and the Hosmer-Lemeshow test (22). The clinical utility of the different models was assessed using decision curve analysis (DCA). The workflow of our study is shown in **Figure 1**.

### 2.7 Statistical analysis

Statistical analyses were performed using SPSS software (version 26.0) and R software (version 4.2.2). Continuous variables were presented as either mean ± standard deviation or median (interquartile range), while categorical variables were expressed as percentages. For continuous variables, comparisons were made using independent samples t-tests or the Wilcoxon rank-sum test. Categorical variables were compared using the χ2 test or Fisher’s exact test. The DeLong test was used to assess statistical differences in the AUCs of the models. The nomogram, calibration curve, Hosmer-Lemeshow test and DCA were calculated using R (version 4.2.2, http://www.r-project.org). A two-tailed P value of less than 0.050 was considered statistically significant.

## 3 Results

### 3.1 Patient characteristics

The distribution of characteristics across the dataset is shown in **Table 1**. Gender and histological type showed significant differences between the EGFR wild-type and EGFR mutation groups in both the training (P < 0.001, P < 0.001, respectively) and validation groups (P = 0.019, P = 0.016, and P = 0.015, respectively). Patients with never smoking, elevated CEA levels and specific CT radiological features (spiculation, pleural indentation and air bronchogram) were more likely to have EGFR mutations in the training group (P < 0.001, P = 0.023, P = 0.036, P = 0.003 and P = 0.041, respectively). However, these differences were not statistically significant in the validation group (all P > 0.05). Compared to the wild-type group, lesion size (MTD and GTV) and all metabolic parameters were lower in the EGFR mutation group. However, with the exception of SUVmean (training group P = 0.020, validation group P = 0.005), no other significant differences were observed between the two cohorts (all P > 0.05).

**Table 1:** Patient characteristics.

Characteristics	Training group			Validation group			P value^c^
	EGFR mutant (n=147)	EGFR wild-type (n=103)	P value^a^	EGFR mutant (n=43)	EGFR wild-type (n=20)	P value^b^	
Age, years, Mean ± SD	60.30 ± 10.11	62.49 ± 11.28	0.111	58.30 ± 10.88	66.15 ± 7.16	0.005	0.787
Gender, n (%)			<0.001			0.019	0.946
Male	73 (49.66%)	75 (72.82%)		21 (48.84%)	16 (80.00%)		
Female	74 (50.34%)	28 (27.18%)		22 (51.16%)	4 (20.00%)		
Smoking, n (%)			<0.001			0.08	0.639
Never smoker	122 (82.99%)	60 (58.25%)		33 (76.74%)	11 (55.00%)		
Current or former smoker	25 (17.01%)	43 (41.75%)		10 (23.26%)	9 (45.00%)		
Tumor location, n (%)			0.517			0.717	0.251
Central	16 (10.88%)	14 (13.59%)		7 (16.28%)	4 (20.00%)		
Peripheral	131 (89.12%)	89 (86.41%)		36 (83.72%)	16 (80.00%)		
Lobe, n (%)			0.012			0.074	0.474
RU	46 (31.29%)	42 (40.78%)		14 (32.56%)	9 (45.00%)		
RM	10 (6.80%)	4 (3.88%)		6 (13.95%)	1 (5.00%)		
RL	23 (15.65%)	29 (28.16%)		10 (23.26%)	2 (10.00%)		
LU	44 (29.93%)	19 (18.45%)		12 (27.91%)	4 (20.00%)		
LL	24 (16.33%)	9 (8.74%)		1 (2.33%)	4 (20.00%)		
Clinical stage, n (%)			0.016			0.066	0.155
I	23 (15.65%)	14 (13.59%)		2 (4.65%)	3 (15.00%)		
II	14 (9.52%)	15 (14.56%)		3 (6.98%)	1 (5.00%)		
III	20 (13.61%)	28 (27.18%)		9 (20.93%)	9 (45.00%)		
IV	90 (61.22%)	46 (44.66%)		29 (67.44%)	7 (35.00%)		
T stage, n (%)			0.033			0.549	0.65
T1	31 (21.09%)	38 (36.89%)		15 (34.88%)	6 (30.00%)		
T2	67 (45.58%)	33 (32.04%)		13 (30.23%)	7 (35.00%)		
T3	18 (12.24%)	14 (13.59%)		4 (9.30%)	4 (20.00%)		
T4	31 (21.09%)	18 (17.48%)		11 (25.58%)	3 (15.00%)		
N stage, n (%)			0.69			0.096	0.051
N0	55 (37.41%)	36 (34.95%)		7 (16.28%)	7 (35.00%)		
N1-3	92 (62.59%)	67 (65.05%)		36 (83.72%)	13 (65.00%)		
M stage, n (%)			0.01			0.015	0.696
M0	57 (38.78%)	57 (55.34%)		14 (32.56%)	13 (65.00%)		
M1	90 (61.22%)	46 (44.66%)		29 (67.44%)	7 (35.00%)		
CEA levels, n (%)			0.023			0.972	0.944
Normal	49 (33.33%)	49 (47.57%)		17 (39.53%)	8 (40.00%)		
Abnormal	98 (66.67%)	54 (52.43%)		26 (60.47%)	12 (60.00%)		
SCC levels, n (%)			0.345			0.055	0.664
Normal	116 (78.91%)	76 (73.79%)		37 (86.05%)	13 (65.00%)		
Abnormal	31 (21.09%)	27 (26.21%)		6 (13.95%)	7 (35.00%)		
CYFRA21-1 levels, n (%)			0.683			0.25	0.641
Normal	59 (40.14%)	44 (42.72%)		17 (39.53%)	11 (55.00%)		
Abnormal	88 (59.86%)	59 (57.28%)		26 (60.47%)	9 (45.00%)		
Histologic_type, n (%)			<0.001			0.016	0.405
Adenocarcinoma	143 (97.28%)	80 (77.67%)		42 (97.67%)	16 (80.00%)		
Adenosquamous carcinoma	4 (2.72%)	3 (2.91%)		0 (0.00%)	0 (0.00%)		
Squamous cell carcinoma	0 (0.00%)	20 (19.42%)		1 (2.33%)	4 (20.00%)		
Lobulation, n (%)			0.611			0.313	0.459
Yes	138 (93.88%)	95 (92.23%)		40 (93.02%)	17 (85.00%)		
No	9 (6.12%)	8 (7.77%)		3 (6.98%)	3 (15.00%)		
Spiculation, n (%)			0.036			0.087	0.084
Yes	65 (44.22%)	32 (31.07%)		25 (58.14%)	7 (35.00%)		
No	82 (55.78%)	71 (68.93%)		18 (41.86%)	13 (65.00%)		
Pleural indentation, n (%)			0.003			0.061	0.399
Yes	87 (59.18%)	41 (39.81%)		28 (65.12%)	8 (40.00%)		
No	60 (40.82%)	62 (60.19%)		15 (34.88%)	12 (60.00%)		
Vacuole sign, n (%)			0.336			0.157	0.558
Yes	13 (8.84%)	13 (12.62%)		2 (4.65%)	3 (15.00%)		
No	134 (91.16%)	90 (87.38%)		41 (95.35%)	17 (85.00%)		
Cavity sign, n (%)						0.492	0.081
Yes	10 (6.80%)	9 (8.74%)	0.57	1 (2.33%)	0 (0.00%)		
No	137 (93.20%)	94 (91.26%)		42 (97.67%)	20 (100.00%)		
Vessel convergence, n (%)			0.389			0.961	0.773
Yes	37 (25.17%)	31 (30.10%)		11 (25.58%)	5 (25.00%)		
No	110 (74.83%)	72 (69.90%)		32 (74.42%)	15 (75.00%)		
Air bronchogram, n (%)			0.041			0.287	0.84
Yes	30 (20.41%)	11 (10.68%)		9 (20.93%)	2 (10.00%)		
No	117 (79.59%)	92 (89.32%)		34 (79.07%)	18 (90.00%)		
Calcification, n (%)			0.903			0.556	0.558
Yes	15 (10.20%)	11 (10.68%)		4 (9.30%)	1 (5.00%)		
No	132 (89.80%)	92 (89.32%)		39 (90.70%)	19 (95.00%)		
MTD (cm), M (Q_1_,Q_3_)	3.48 (2.70, 4.29)	3.59 (2.54, 4.85)	0.763	3.18 (2.51, 4.19)	3.23 (2.67, 5.27)	0.483	0.328
GTV (cm^3^), M (Q_1_,Q_3_)	11.78 (5.70, 21.83)	12.49 (4.43, 29.41)	0.687	7.68 (5.27, 16.33)	12.60 (4.87, 44.18)	0.38	0.433
SUVmax, M (Q_1_,Q_3_)	11.55 (8.43, 13.89)	12.23 (8.78, 16.00)	0.06	9.02 (7.06, 14.16)	12.54 (8.79, 17.50)	0.053	0.552
SUVpeak, M (Q_1_,Q_3_)	8.33 (5.59, 10.46)	8.76 (6.06, 11.60)	0.15	7.05 (5.10, 10.03)	9.45 (5.94, 13.65)	0.146	0.507
SUVmean, M (Q_1_,Q_3_)	6.16 (4.83, 7.23)	6.78 (5.09, 8.00)	0.02	5.17 (4.15, 7.05)	7.55 (5.57, 9.05)	0.005	0.656
MTV (cm^3^), M (Q_1_,Q_3_)	7.43 (3.60, 13.67)	9.08 (2.81, 20.05)	0.28	4.54 (3.18, 10.23)	9.25 (3.28, 28.02)	0.348	0.344
TLG (g), M (Q_1_,Q_3_)	44.98 (17.92, 96.68)	55.06 (16.26, 182.81)	0.115	23.39 (14.04, 61.09)	60.06 (25.71, 248.45)	0.095	0.323

Data are expressed as n (%) or median [interquartile range] or mean ± standard deviation. EGFR, epidermal growth factor receptor; RU, right upper; RM, right middle; RL, right lower; LU, left upper; LL, left lower; CEA, carcinoembryonic antigen; SCC, squamous cell carcinoma antigen; CYFRA21-1, cytokeratin 19 fragment antigen21-1; MTD, maximum tumor diameter; GTV, gross tumor volume; SUVmax, maximum standard uptake value; SUVpeak, peak standard uptake value; SUVmean, mean standard uptake value; MTV, metabolic tumor volume; TLG, total lesion glycolysis.

a EGFR mutant vs. EGER wild-type in training group.

b EGFR mutant vs. EGER wild-type in validation group.

c Training group vs. validation group.

### 3.2 Feature selection and model performance evaluation

**Figure 2:**
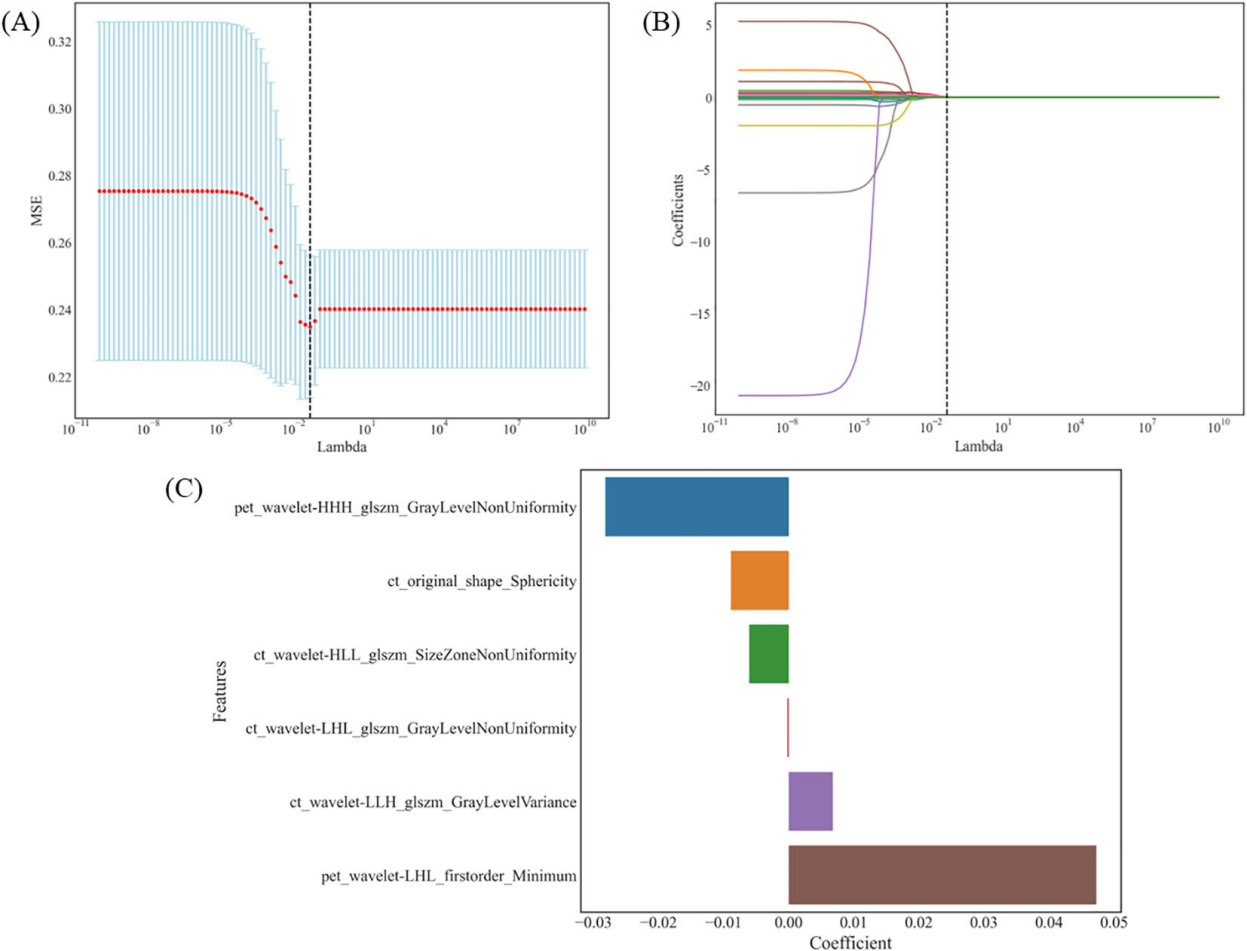
The LASSO algorithm and tenfold cross-validation for the selection of radiomic features. **(A)** MSE trend associated with the change in λ: λ is selected by 10-fold cross-validation. When λ=0.0486, the MSE is minimum and the Lasso regression model shows the best performance. **(B)** Coefficient trend of each feature along with λ: 6 radiomic features with non-zero coefficients were selected in the final model. **(C)** The coefficient values of these radiomic features in the LASSO model. LASSO, least absolute shrinkage and selection operator; MSE, mean square error; λ, lambda.

Finally, six optimal features were identified using the LASSO algorithm and tenfold cross-validation, consisting of four CT and two PET radiomics features. These features included: CT-original shape sphericity, CT-wavelet-HLL_glszm_SizeZoneNonUniformity, CT-wavelet-LHL_glszm_GrayLevelNonUniformity, CT-wavelet-LLH_glszm_GrayLevelVariance, PET-wavelet-HHH_glszm_GrayLevelNonUniformity, and PET-wavelet-LHL_firstorder_Minimum (as shown in **Figure 2**). The intraobserver and interobserver ICC of these six radiomics features were 0.9055-0.9979 and 0.9645-0.9979, respectively. In this study, four classifiers were used to construct radiomics-based models: LR, RF, SVM and XGBoost. The detailed performance metrics for each model are shown in **Table 2**. The DeLong test revealed that the RF model’s AUC surpassed those of the LR and SVM models with statistical significance (P < 0.001) and was comparable to the XGBoost model in the training group. In the validation group, RF exhibited the highest AUC, albeit without a significant difference between the groups. Consequently, the RF model demonstrated consistent and superior predictive performance, deeming it the most fitting radiomics model for this study. Separately, 4 clinical variables (smoking history, sex, adenocarcinoma and squamous cell carcinoma) and 3 radiological features (air bronchus sign, pleural indentation sign, and spiculation sign) were obtained through multi-step screening to establish corresponding models. The clinical model for distinguishing EGFR mutations, achieved an AUC of 0.711 (95% CI, 0.645-0.776) in the training group and 0.758 (95% CI, 0.627-0.890) in the validation group. The performance of radiological model was moderate, with AUC values of 0.632 (95% CI, 0.564-0.699) and 0.677 (95% CI, 0.531-0.822) in the training and validation groups, respectively.

**Table 2:** The diagnostic performance of each model in identifying EGFR mutations.

Model	AUC (95%CI)	Sensitivity (95%CI)	Specificity(95%CI)	PPV (95%CI)	NPV (95%CI)	Accuracy (95%CI)
Training group						
LR	0.659 (0.591-0.728)	0.551 (0.471-0.631)	0.699 (0.610-0.788)	0.723 (0.640-0.806)	0.522 (0.438-0.605)	0.612 (0.552-0.672)
RF	0.785 (0.726-0.844)	0.755 (0.686-0.825)	0.709 (0.621-0.796)	0.787 (0.720-0.855)	0.670 (0.581-0.758)	0.736 (0.681-0.791)
SVM	0.686 (0.619-0.753)	0.796 (0.731-0.861)	0.495 (0.399-0.592)	0.692 (0.623-0.762)	0.630 (0.524-0.735)	0.672 (0.614-0.730)
XGBoost	0.794 (0.737-0.851)	0.701 (0.627-0.775)	0.777 (0.696-0.857)	0.817 (0.750-0.885)	0.645 (0.561-0.729)	0.732 (0.677-0.787)
Clinical Model	0.711 (0.645-0.776)	0.762 (0.693-0.831)	0.534 (0.438-0.630)	0.700 (0.629-0.771)	0.611 (0.510-0.712)	0.668 (0.610-0.726)
Radiological Model	0.632 (0.564-0.699)	0.748 (0.678-0.818)	0.456 (0.360-0.553)	0.663 (0.591-0.735)	0.560 (0.453-0.666)	0.628 (0.568-0.688)
Combined Model	0.872 (0.829-0.915)	0.796 (0.731-0.861)	0.786 (0.707-0.866)	0.842 (0.781-0.902)	0.730 (0.647-0.812)	0.792 (0.742-0.842)
Validation group						
LR	0.710 (0.579-0.842)	0.535 (0.386-0.684)	0.800 (0.625-0.975)	0.852 (0.718-0.986)	0.444 (0.282-0.607)	0.619 (0.499-0.739)
RF	0.776 (0.662-0.889)	0.698 (0.560-0.835)	0.650 (0.441-0.859)	0.811 (0.685-0.937)	0.500 (0.308-0.692)	0.683 (0.568-0.797)
SVM	0.734 (0.611-0.856)	0.767 (0.641-0.894)	0.500 (0.281-0.719)	0.767 (0.641-0.894)	0.500 (0.281-0.719)	0.683 (0.568-0.797)
XGBoost	0.724 (0.601-0.848)	0.558 (0.410-0.707)	0.850 (0.694-1.000)	0.889 (0.770-1.000)	0.472 (0.309-0.635)	0.651 (0.533-0.769)
Clinical Model	0.758 (0.627-0.890)	0.721 (0.587-0.855)	0.750 (0.560-0.940)	0.861 (0.748-0.974)	0.556 (0.368-0.743)	0.730 (0.621-0.840)
Radiological Model	0.677 (0.531-0.822)	0.721 (0.587-0.855)	0.600 (0.385-0.815)	0.795 (0.668-0.922)	0.500 (0.300-0.700)	0.683 (0.568-0.797)
Combined Model	0.831 (0.723-0.940)	0.698 (0.560-0.835)	0.800 (0.625-0.975)	0.882 (0.774-0.991)	0.552 (0.371-0.733)	0.730 (0.621-0.840)

AUC, area under the curve; PPV, positive predictive value; NPV, negative predictive value; LR, logistic regression; RF, random forest; SVM, support vector machine; XGBoost, extreme gradient boosting.

### 3.3 Establishment and evaluation of the nomogram prediction model

**Figure 3:**
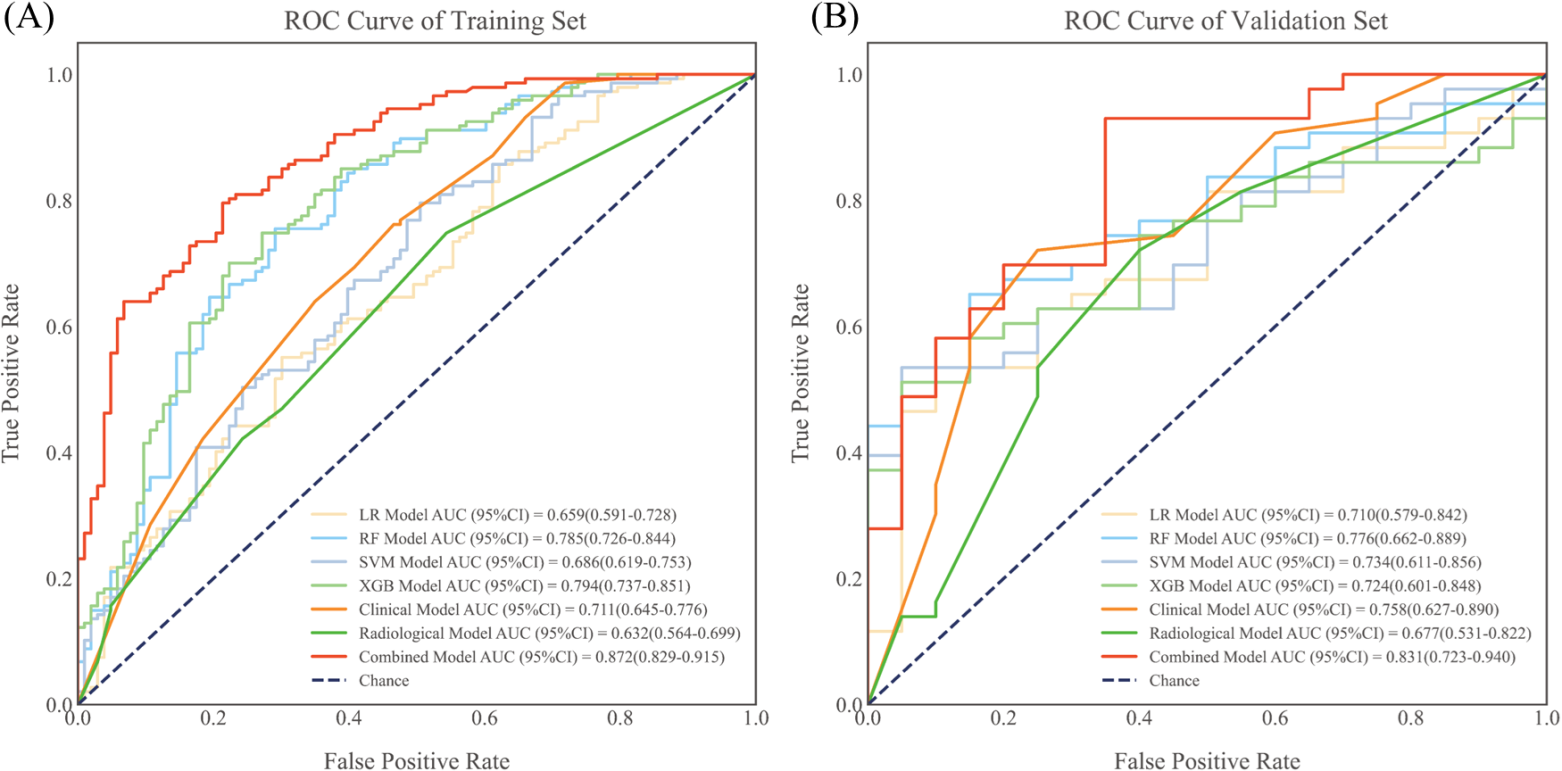
ROC curves for each prediction model in the training group **(A)** and validation group **(B)**. All model results were evaluated using quintuple cross-validation. ROC, receiver operating characteristic; AUC, area under the curve; LR, logistic regression; RF, random forest; SVM, support vector machine; XGBoost, extreme gradient boosting.

**Figure 4:**
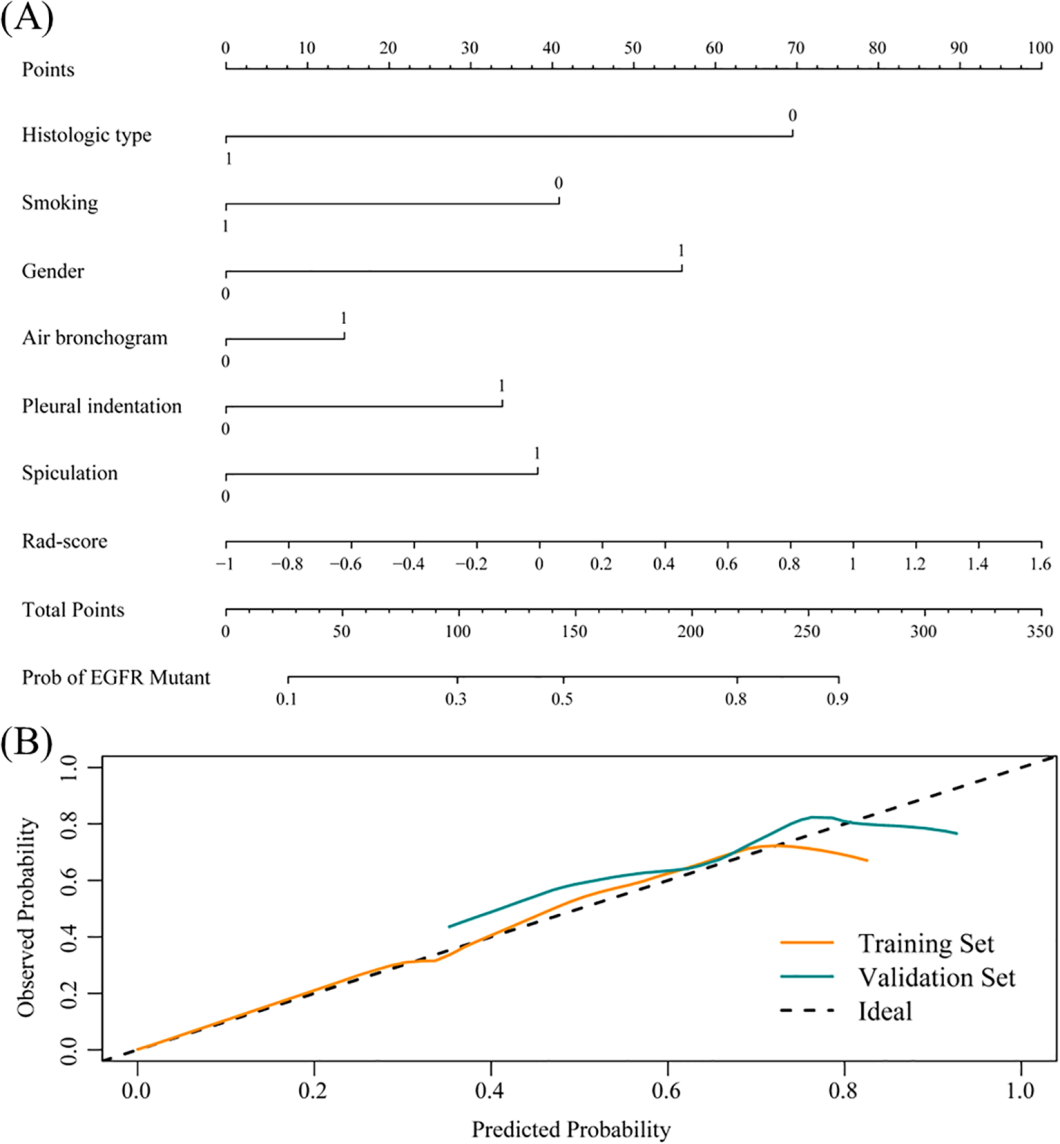
Nomogram evolution and performance. **(A)** Nomogram based on the combined model. Histological type: 1 represents SCC, 0 represents ADC; Smoking: 1 represents current or former smoker, 0 represents never smoker; Gender: 1 represents female, 0 represents male; Radiological signs: 1 represents yes, 0 represents no. **(B)** Calibration curve of the nomogram in the training and validation groups.

**Figure 5:**
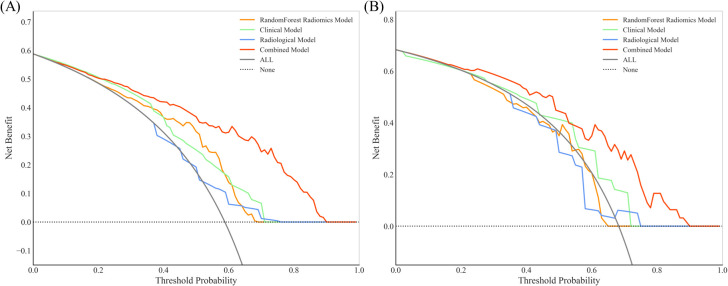
Decision curve analysis (DCA) for RF radiomic, clinical, radiological and combined models in the training group **(A)** and validation group **(B)**. The combined model for EGFR prediction added more value than the use of the treat-all scheme for threshold probabilities >20% in both the training and validation groups.

To improve predictive accuracy, we combined radiomic, clinical and radiological features to create a combined model. This model achieved an AUC of 0.872 (95% CI, 0.829-0.915) and 0.831 (95% CI, 0.723-0.940) in the training and validation groups, respectively, as shown in **Table 3** and **Figure 3**. In the training group, the AUC of the combined model outperformed that of the RF radiomics model (P = 0.010), the clinical model (P < 0.001) and the radiological model (P < 0.001). In the validation group, it outperformed the other three models, with a significant difference noted only against the radiological model (P = 0.017). Overall, the combined model showed superior predictive ability. As a result, we created an individualized nomogram in the validation group, which provides an intuitive visualization of the prediction results and their influencing factors (**Figure 4A**). Among the predictors, Rad-score was the most influential in predicting EGFR mutation. The Hosmer-Lemeshow test confirmed the accuracy of the combined model in both the training (χ2 = 7.3975, P = 0.495) and validation groups (χ2 = 9.8997, P = 0.272). Calibration curves further highlighted the agreement between observed and predicted results (**Figure 4B**). Decision curve analysis (DCA), shown in **Figure 5**, revealed that the area under the curve of the combined model outperformed other models, highlighting its superior clinical utility.

**Table 3:** The Delong test for RF radiomic, clinical, radiological and combined model.

Model	AUC (95%CI)	Statistics	*P* Value
Training set			
RF	0.785 (0.726-0.844)	-2.55681	0.010
Clinical Model	0.711 (0.645-0.776)	-6.61029	<0.001
Radiological Model	0.632 (0.564-0.699)	-7.92496	<0.001
Combined Model	0.872 (0.829-0.915)	Ref	Ref
Validation set			
RF	0.776 (0.662-0.889)	-1.34423	0.179
Clinical Model	0.758 (0.627-0.890)	-1.66593	0.096
Radiological Model	0.677 (0.531-0.822)	-2.37707	0.017
Combined Model	0.831 (0.723-0.940)	Ref	Ref

Using the Combined Model as a reference.

## 4 Discussion

Given the clear benefit of TKIs for NSCLC patients with an EGFR mutation, accurate detection of EGFR gene mutation status becomes critical for informed clinical treatment decisions. In this retrospective study, we developed individualized nomograms integrating ^18^F-FDG PET/CT radiomics, radiological and clinical features to provide a non-invasive approach to predict EGFR mutation status in NSCLC patients.

Previous research has shown that females, adenocarcinoma patients and non-smokers are more likely to have EGFR mutations (22, 23), a finding consistent with our results. By integrating these variables, our clinical model achieved AUCs of 0.711 and 0.758 in the training and validation groups, respectively. While numerous studies have investigated the relationship between ^18^F-FDG uptake and EGFR mutation status in NSCLC, their results have been inconsistent. Our analysis of PET metabolic parameters between the EGFR mutation and wild-type groups (detailed in **Supplementary Table S1**) is largely consistent with previous studies (24, 25). We observed minimal correlation between ^18^F-FDG uptake and EGFR mutation status in both groups, leading us to exclude PET metabolic parameters from our radiological model. Notably, while several studies have suggested that a higher SUVmax indicates the presence of an EGFR mutation, others have found no association between ^18^F-FDG uptake and EGFR mutation status (26, 27). Such discrepancies may be due to differences in sample size, sample characteristics, or ROI selection and measurement methods. In conclusion, PET metabolic parameters appear to have limited predictive value for EGFR mutations.

The integration of CT equipment into PET/CT scanners enhances the clarity of morphological features, potentially improving the diagnostic accuracy for NSCLC patients. Our study confirmed that CT morphological features such as air bronchial sign, pleural indentation sign and spicule sign were associated with an increased risk of EGFR mutation, which is consistent with previous studies (12, 28, 29). In addition, other studies have found that ground glass nodules (GGNs), which include both pure and mixed ground glass nodules, are often indicative of EGFR mutations in NSCLC patients (30, 31). Given the challenges in delineating GGNs on PET images, they were excluded from our study. Notably, our radiological model based on CT morphological features showed moderate predictive performance, with AUC values of 0.632 and 0.677 in the training and validation groups, respectively.

Research on radiomics for EGFR prediction is growing rapidly, with many studies confirming its feasibility and potential benefits. In our study, we used four machine learning classifiers (LR, RF, SVM and XGBoost) to construct an EGFR mutation prediction model using six optimal features refined by a four-step dimensionality reduction process. The AUC values for these models ranged from 0.659 to 0.794 in the training group and from 0.710 to 0.776 in the validation group. Given the relative stability and commendable predictive performance of the RF model, with AUC values of 0.785 and 0.776 in the training and validation groups respectively, it was selected for further research. Although the difference wasn’t statistically significant, we expect this to be resolved by increasing the sample size. Introduced by Breiman in 2001, RF is an ensemble learning method suitable for both classification and regression. It uses a collection of decision trees to create a diversified prediction model. Due to its robust predictive accuracy, resistance to overfitting, ability to model complex non-linear relationships and interpretability, RF has gained traction in biomedical engineering (32–34). Wang et al. (35) demonstrated that an RF model based on preoperative CT radiomics features could detect EGFR mutations in lung adenocarcinoma patients, achieving AUC values of 0.70 and 0.64 for the training and validation groups, respectively. Gu et al. highlighted the superior performance of an RF-based radiomics classifier (AUC=0.776) in predicting Ki-67 expression levels in NSCLC patients (36). Some studies (24, 26, 37) have suggested that radiomics signatures from ^18^F-FDG PET/CT images provide better EGFR mutation predictions than those from stand-alone CT or conventional PET images. Recent studies typically report AUC values between 0.57 and 0.86 when relying on PET/CT radiomics features (16, 24, 38, 39). While factors such as image data sources, spatial resolution, post-processing, model algorithms and data size can introduce variability, the collective body of work, including our study, underscores the potential of radiomics-based machine learning models for EGFR mutation prediction.

In our study, PET and CT images were filtered and pre-processed prior to feature extraction. This step is critical because it minimizes image acquisition errors and ensures that the results are both stable and reliable. Our results underline that the final six radiomics features are highly reliable. Even when different machine learning algorithms are used to construct models using these features, the resulting models consistently show commendable predictive performance. Notably, five of the six radiomics features were wavelet features, highlighting the central role of features derived from wavelet-filtered images in the radiomics model. Wavelet transform, a widely used method for noise reduction, data smoothing and filtering, is excellent at revealing specific patterns hidden in large datasets. By capturing tumor heterogeneity, wavelet features potentially improve the predictive power of the model (40). Similarly, Zhang et al. (41) found that seven out of twelve wavelet-transformed features correlated with EGFR mutations. This suggests that texture and high-dimensional features may have a more robust association with EGFR mutation status.

Although the RF radiomics model in our study showed slightly better predictive power than the clinical and radiological models, reliance on it alone for clinical applications may be limited. Zhang et al. (38) found that a model combining PET/CT radiomics with clinical features (gender and smoking history) outperformed a model based on PET/CT radiomics alone, with AUC values of 0.86 vs 0.79 in the training group and 0.87 vs 0.85 in the validation group. Another study constructed an integrated model using CT radiomics, CT radiological features and clinical features to predict EGFR mutations in adenocarcinoma patients, achieving AUCs of 0.849 and 0.835 in the training and validation groups, respectively (41). Chang et al. (37) also showed that a combined model integrating PET/CT radiomics with CT morphological features was more effective in predicting EGFR mutations in lung adenocarcinoma than a model based on PET/CT radiomics alone (AUC: 0.84 vs. 0.76 in the training group and 0.81 vs. 0.75 in the validation group). In our study, the combined model incorporating rad-score, clinical and radiological features achieved AUCs of 0.872 and 0.831 in the training and validation groups, respectively, outperforming the stand-alone clinical and radiological models. To further improve the clinical utility, we developed a radiomics-based nomogram that integrates the Rad score with the aforementioned clinical and radiological features to provide a visualized prediction. Decision curve analysis (DCA) further validated the clinical applicability of this nomogram.

Our study has several limitations that need to be considered. First, it is a single-center retrospective study with a relatively small sample size. A larger sample size can enhance research reliability, interpretability, and generalization, while mitigating selection bias. To further validate and improve model performance, we plan to expand the sample size or collaborate with multicenter data for both single-center and external validations in future work. Second, the use of manual and semi-automated outlining methods introduces the possibility of human error. These methods may also lack the repeatability seen with fully automated outlining. Third, while our study provides an initial exploration of radiomics using four different classifiers, the optimal feature selection method and machine learning algorithm for specific applications remains a matter of debate. Future research will combine radiomics with deep learning to achieve fully automated analysis of the entire process from tumor segmentation to prediction, and improve prediction efficiency (AUC > 0.9) to enhance its clinical applicability.

## 5 Conclusions

In conclusion, the combination of radiomics and machine learning using ^18^F-FDG PET/CT images offers a promising approach to identify EGFR mutation status in NSCLC patients. The integration of clinical and radiological features with the Rad score further improves the predictive accuracy. Radiomics-based nomograms provide a valuable, non-invasive and visually intuitive tool for screening patients with EGFR mutations in a clinical setting.

## Data Availability

The original contributions presented in the study are included in the article/**Supplementary Material**. Further inquiries can be directed to the corresponding authors.
