# Supplementary material for: Prediction of EGFR mutations in non-small cell lung cancer: a nomogram based on 18F-FDG PET and thin-section CT radiomics with machine learning
Source: Front Oncol. 2025 Apr 2;15:1510386. doi: 10.3389/fonc.2025.1510386 (PMC11999825; doi:10.3389/fonc.2025.1510386)
Supplement: Supplementary file 1 [file Table1.docx]

Supplementary Material

# Supplementary Table 1. Patient characteristics between EGFR mutant and wild-type patients

| Characteristics | Total (n=313) | EGFR status | | Statistics | P value |
| --- | --- | --- | --- | --- | --- |
|  |  | EGFR mutant type (n=190) | EGFR wild type (n=123) |  |  |
| Age, years, Mean±SD | 61.12±10.63 | 59.85±10.33 | 63.08±10.80 | t=-2.649 | 0.008 |
| Gender, n (%) |  |  |  | χ2=18.557 | <0.001 |
| Male | 185 (59.11%) | 94 (49.47%) | 91 (73.98%) |  |  |
| Female | 128 (40.89%) | 96 (50.53%) | 32 (26.02%) |  |  |
| Smoking, n (%) |  |  |  | χ2=21.171 | <0.001 |
| Never smoker | 226 (72.20%) | 155 (81.58%) | 71 (57.72%) |  |  |
| Current or former smoker | 87 (27.80%) | 35 (18.42%) | 52 (42.28%) |  |  |
| Tumor location, n (%) |  |  |  | χ2=0.419 | 0.517 |
| Central | 41 (13.10%) | 23 (12.11%) | 18 (14.63%) |  |  |
| Peripheral | 272 (86.90%) | 167 (87.89%) | 105 (85.37%) |  |  |
| Lobe, n (%) |  |  |  | χ2=10.257 | 0.036 |
| RU | 111 (35.46%) | 60 (31.58%) | 51 (41.46%) |  |  |
| RM | 21 (6.71%) | 16 (8.42%) | 5 (4.07%) |  |  |
| RL | 64 (20.45%) | 33 (17.37%) | 31 (25.20%) |  |  |
| LU | 79 (25.24%) | 56 (29.47%) | 23 (18.70%) |  |  |
| LL | 38 (12.14%) | 25 (13.16%) | 13 (10.57%) |  |  |
| Clinical stage, n (%) |  |  |  | χ2=14.156 | 0.003 |
| Ⅰ | 42 (13.42%) | 25 (13.16%) | 17 (13.82%) |  |  |
| Ⅱ | 33 (10.54%) | 17 (8.95%) | 16 (13.01%) |  |  |
| Ⅲ | 66 (21.09%) | 29 (15.26%) | 37 (30.08%) |  |  |
| Ⅳ | 172 (54.95%) | 119 (62.63%) | 53 (43.09%) |  |  |
| T stage, n (%) |  |  |  | χ2=6.745 | 0.081 |
| T1 | 90 (28.75%) | 46 (24.21%) | 44 (35.77%) |  |  |
| T2 | 120 (38.34%) | 80 (42.11%) | 40 (32.52%) |  |  |
| T3 | 40 (12.78%) | 22 (11.58%) | 18 (14.63%) |  |  |
| T4 | 63 (20.13%) | 42 (22.11%) | 21 (17.07%) |  |  |
| N stage, n (%) |  |  |  | χ2=0.181 | 0.67 |
| N0 | 105 (33.55%) | 62 (32.63%) | 43 (34.96%) |  |  |
| N1-3 | 208 (66.45%) | 128 (67.37%) | 80 (65.04%) |  |  |
| M stage, n (%) |  |  |  | χ2=11.519 | <0.001 |
| M0 | 141 (45.05%) | 71 (37.37%) | 70 (56.91%) |  |  |
| M1 | 172 (54.95%) | 119 (62.63%) | 53 (43.09%) |  |  |
| CEA levels, n (%) |  |  |  | χ2=4.215 | 0.04 |
| Normal | 123 (39.30%) | 66 (34.74%) | 57 (46.34%) |  |  |
| Abnormal | 190 (60.70%) | 124 (65.26%) | 66 (53.66%) |  |  |
| SCC levels, n (%) |  |  |  | χ2=2.841 | 0.092 |
| Normal | 242 (77.32%) | 153 (80.53%) | 89 (72.36%) |  |  |
| Abnormal | 71 (22.68%) | 37 (19.47%) | 34 (27.64%) |  |  |
| CYFRA21-1 levels, n (%) |  |  |  | χ2=0.682 | 0.409 |
| Normal | 131 (41.85%) | 76 (40.00%) | 55 (44.72%) |  |  |
| Abnormal | 182 (58.15%) | 114 (60.00%) | 68 (55.28%) |  |  |
| Histologic type, n (%) |  |  |  | χ2=36.837 | <0.001 |
| Adenocarcinoma | 281 (89.78%) | 185 (97.37%) | 96 (78.05%) |  |  |
| Adenosquamous carcinoma | 7 (2.24%) | 4 (2.11%) | 3 (2.44%) |  |  |
| Squamous cell carcinoma | 25 (7.99%) | 1 (0.53%) | 24 (19.51%) |  |  |
| Lobulation, n (%) |  |  |  | χ2=0.757 | 0.384 |
| Yes | 290 (92.65%) | 178 (93.68%) | 112 (91.06%) |  |  |
| No | 23 (7.35%) | 12 (6.32%) | 11 (8.94%) |  |  |
| Spiculation, n (%) |  |  |  | χ2=7.559 | 0.006 |
| Yes | 129 (41.21%) | 90 (47.37%) | 39 (31.71%) |  |  |
| No | 184 (58.79%) | 100 (52.63%) | 84 (68.29%) |  |  |
| Pleural indentation, n (%) |  |  |  | χ2=12.813 | <0.001 |
| Yes | 164 (52.40%) | 115 (60.53%) | 49 (39.84%) |  |  |
| No | 149 (47.60%) | 75 (39.47%) | 74 (60.16%) |  |  |
| Vacuole sign, n (%) |  |  |  | χ2=2.188 | 0.139 |
| Yes | 31 (9.90%) | 15 (7.89%) | 16 (13.01%) |  |  |
| No | 282 (90.10%) | 175 (92.11%) | 107 (86.99%) |  |  |
| Cavity sign, n (%) |  |  |  | χ2=0.291 | 0.589 |
| Yes | 20 (6.39%) | 11 (5.79%) | 9 (7.32%) |  |  |
| No | 293 (93.61%) | 179 (94.21%) | 114 (92.68%) |  |  |
| Vessel convergence, n (%) |  |  |  | χ2=0.610 | 0.435 |
| Yes | 84 (26.84%) | 48 (25.26%) | 36 (29.27%) |  |  |
| No | 229 (73.16%) | 142 (74.74%) | 87 (70.73%) |  |  |
| Air bronchogram, n (%) |  |  |  | χ2=5.344 | 0.021 |
| Yes | 52 (16.61%) | 39 (20.53%) | 13 (10.57%) |  |  |
| No | 261 (83.39%) | 151 (79.47%) | 110 (89.43%) |  |  |
| Calcification, n (%) |  |  |  | χ2=0.005 | 0.944 |
| Yes | 31 (9.90%) | 19 (10.00%) | 12 (9.76%) |  |  |
| No | 282 (90.10%) | 171 (90.00%) | 111 (90.24%) |  |  |
| MTD (cm), M (Q_1_,Q_3_) | 3.46 (2.63, 4.42) | 3.38 (2.66, 4.29) | 3.59 (2.55, 4.89) | Z=0.670 | 0.503 |
| GTV (cm^3^), M (Q_1_,Q_3_) | 11.35 (5.25, 23.61) | 10.83 (5.33, 19.41) | 12.50 (4.43, 29.95) | Z=0.790 | 0.43 |
| SUVmax, M (Q_1_,Q_3_) | 11.65 (8.35, 14.88) | 11.54 (8.05, 14.01) | 12.26 (8.72, 16.19) | Z=2.512 | 0.012 |
| SUVpeak, M (Q_1_,Q_3_) | 8.33 (5.56, 10.92) | 7.94 (5.40, 10.34) | 8.78 (6.06, 11.61) | Z=2.045 | 0.041 |
| SUVmean, M (Q_1_,Q_3_) | 6.32 (4.83, 7.53) | 6.03 (4.54, 7.21) | 6.87 (5.23, 8.16) | Z=3.360 | <0.001 |
| MTV (cm^3^), M (Q_1_,Q_3_) | 7.43 (3.21, 16.34) | 6.64 (3.32, 12.54) | 9.08 (2.81, 20.34) | Z=1.430 | 0.153 |
| TLG (g), M (Q_1_,Q_3_) | 46.62 (16.28, 109.09) | 39.14 (16.28, 91.36) | 55.19 (16.47, 188.98) | Z=2.173 | 0.03 |
